# Supplementary material for: Spin-Crossover and Slow Magnetic Relaxation Behavior in Hexachlororhenate(IV) Salts of Mn(III) Complexes [Mn(5-Hal-sal2323)]2[ReCl6] (Hal = Cl, Br)
Source: Int J Mol Sci. 2022 Sep 28;23(19):11449. doi: 10.3390/ijms231911449 (PMC9570138; doi:10.3390/ijms231911449)
Supplement: Supplementary file 1 [file ijms-23-11449-s001.zip › ijms-1904460-supplementary.pdf]

International Journal of
Molecular Sciences

MDPI

ELECTRONIC SUPPORTING INFORMATION (ESI)
for International Journal of Molecular Sciences (MDPI)

Spin-Crossover and Slow Magnetic Relaxation Behavior in Hexachlororhenate(IV) Salts of Mn(III) Complexes $[Mn(5\text{-}Hal\text{-}sal_2323)]_2[ReCl_6]$ (Hal = Cl, Br)

Aleksandra V. Tiunova [1,2], Anna V. Kazakova [2], Denis V. Korchagin [2,*], Gennady V. Shilov [2], Sergey M. Aldoshin [2], Aleksei I. Dmitriev [2], Mikhail V. Zhidkov [2], Konstantin V. Zakharov [3] and Eduard B. Yagubskii [2,*]

[1] Faculty of Fundamental Physical and Chemical Engineering, Lomonosov Moscow State University, 119991 Moscow, Russia
[2] Federal Research Center of Problems of Chemical Physics and Medicinal Chemistry RAS, 142432 Chernogolovka, Russia
[3] Department of Low Temperature Physics and Superconductivity, Lomonosov Moscow State University, 119991 Moscow, Russia
* Correspondence: korden@icp.ac.ru (D.V.K.); yagubski@gmail.com (E.B.Y.)

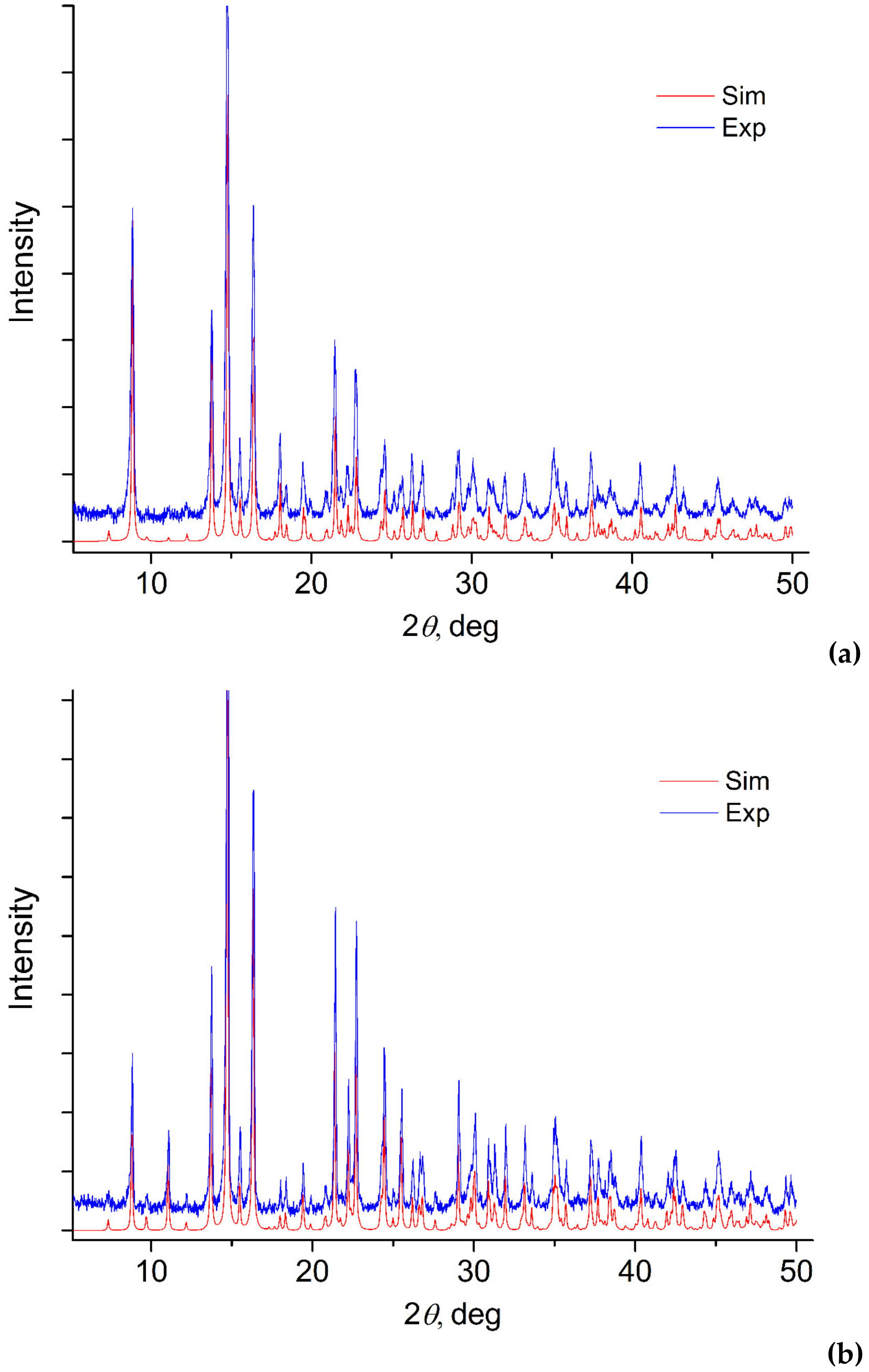

Figure S1. Powder X-ray diffraction pattern of polycrystalline samples of **1** (a) and **2** (b) experimental (blue), and simulated from single crystal data (red).

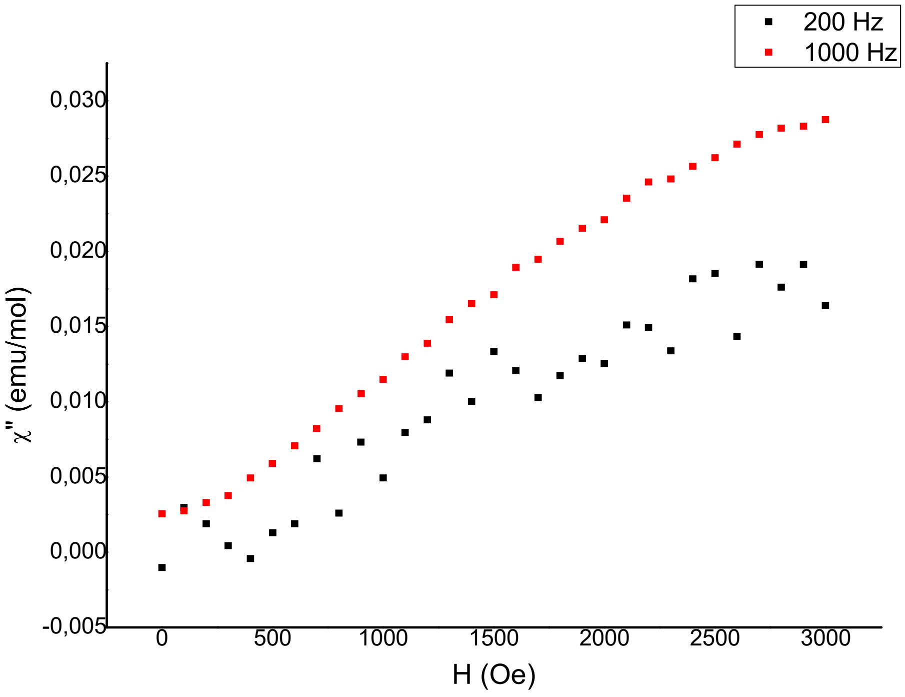

Figure S2. The field-dependence of the χ`` at T=2 K and fixed frequencies of 200 and 1000 Hz ac excitation for complexes **1**.

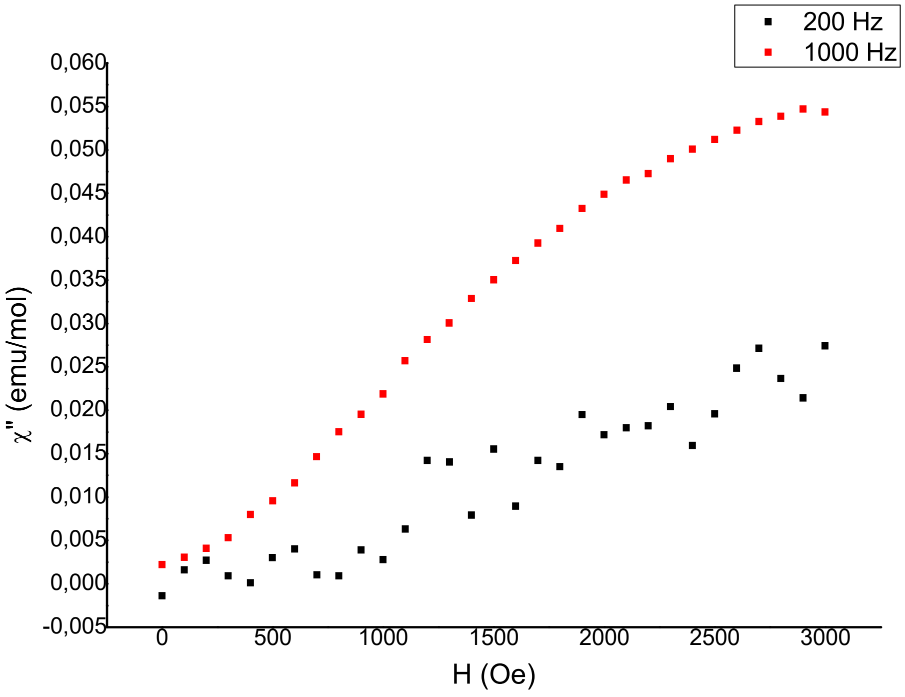

Figure S3. The field-dependence of the χ`` at T=2 K and fixed frequencies of 200 and 1000 Hz ac excitation for complexes **2**.

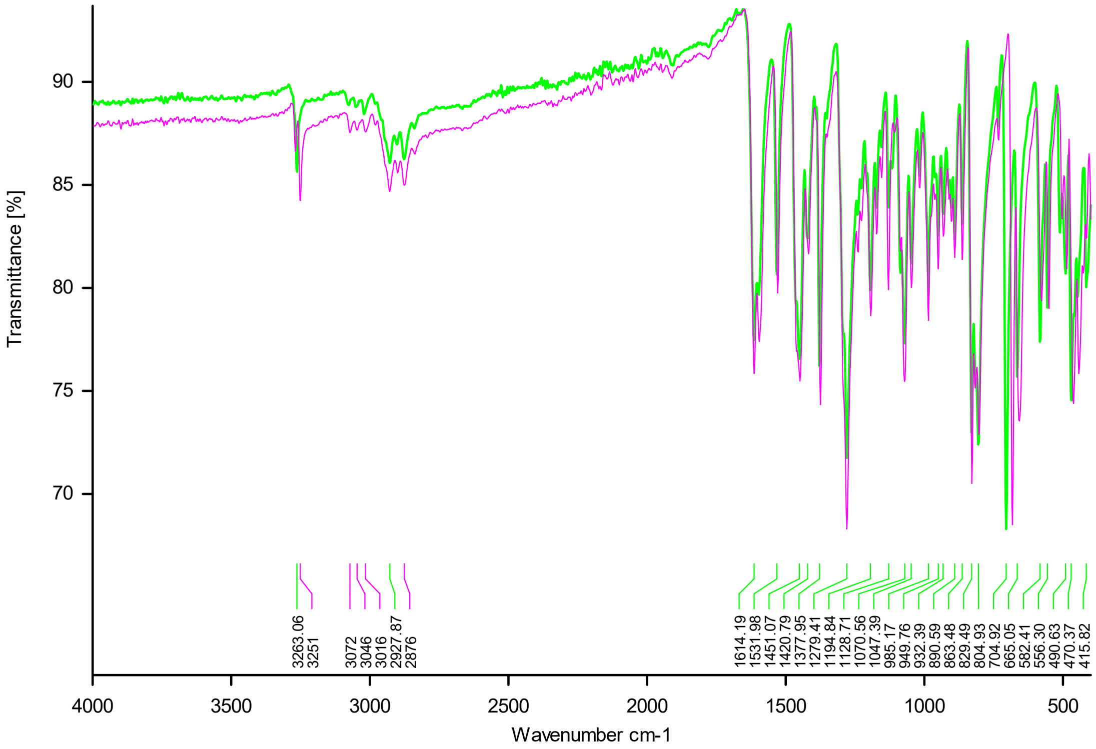

Figure S4. IR-spectra of the complexes **1** (green) and **2** (red).

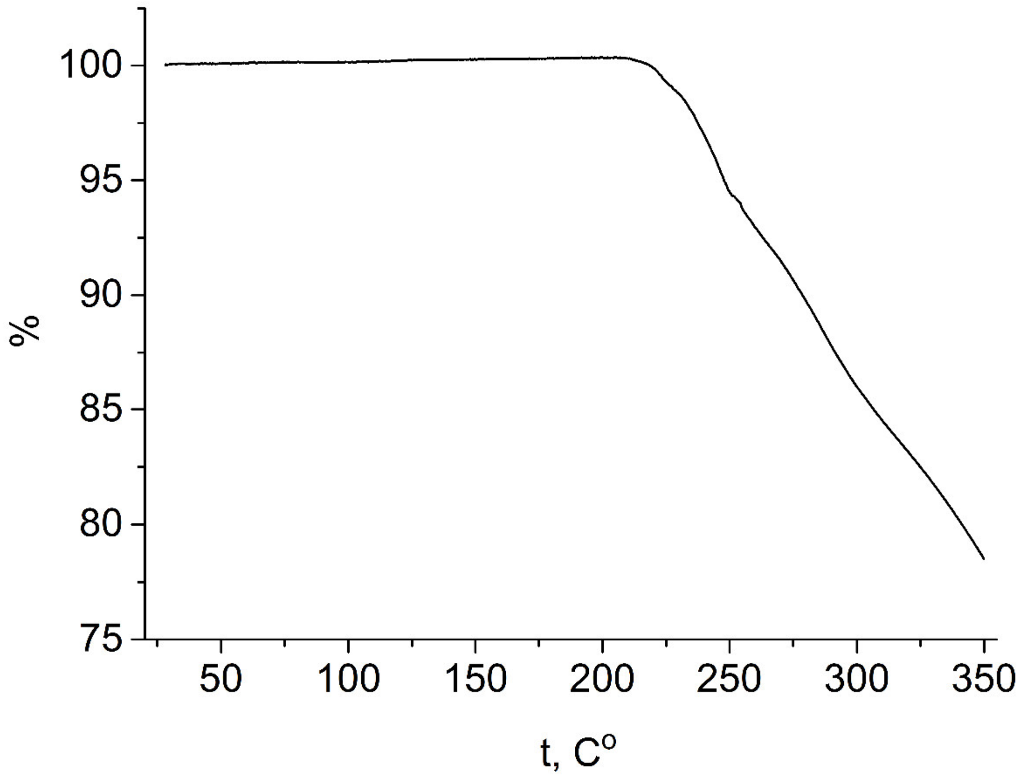

Figure S5. Thermogram of the complex $[Mn(5\text{-}Cl\text{-}sal_2323)]_2(ReCl_6)$ (**1**).

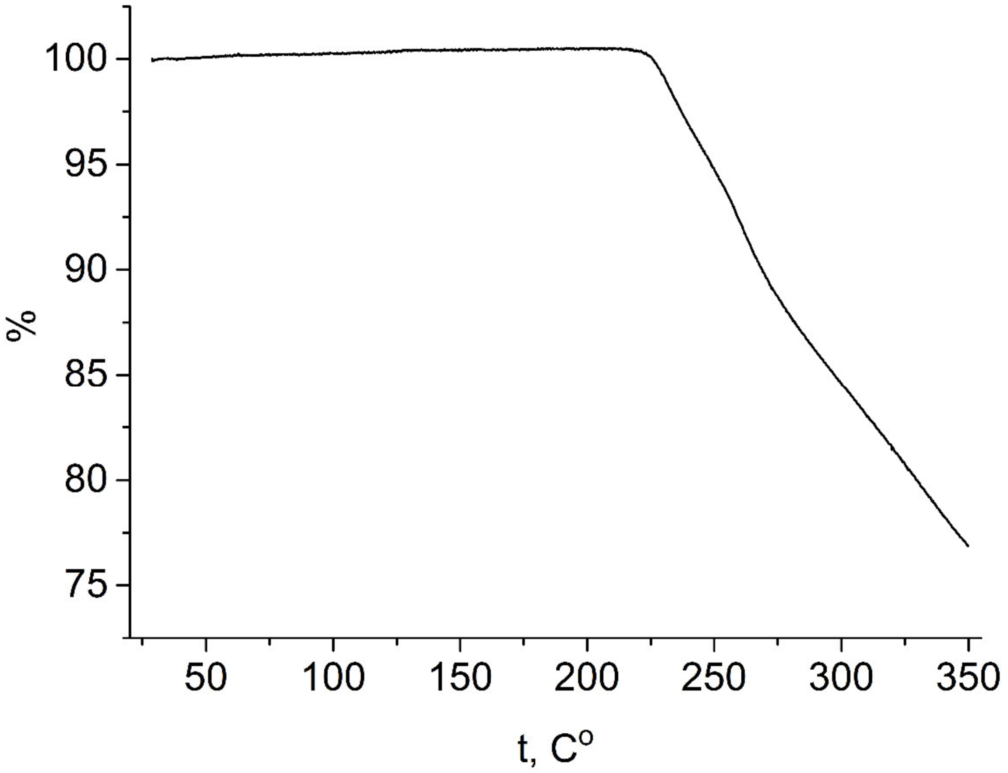

Figure S6. Thermogram of the complex [Mn(5-Br-$sal_2$323)]$_2$($ReCl_6$) (**2**).

Table S1. Crystal data and structure refinement parameters for compound **1**.

Temp., K	100	240	300	353	403	423
Empirical formula	$[C_{22}H_{26}Cl_2N_4O_2Mn]_2^{+}[ReCl_6]^{2-}$					
Molecular weight	1407.51					
Crystal system, space group	monoclinic, *C*2/*c*					
a, Å	18.0559(3)	18.1386(2)	18.1940(3)	18.3010(6)	18.3700(4)	18.3851(5)
b, Å	15.8002(2)	15.8993(2)	15.9448(2)	15.9594(5)	16.0449(4)	16.0470(4)
c, Å	17.9910(3)	18.0532(2)	18.0884(2)	18.1232(5)	18.1805(4)	18.1896(5)
β, °	90.267(1)	90.194(1)	90.008(1)	90.249(3)	90.499(2)	90.575(2)
Volume, Å^3	5132.5(1)	5206.4(1)	5247.5(1)	5293.3(3)	5358.4(2)	5366.1(2)
Z, $\varrho_{calc.}$, g/cm^3	4, 1.822	4, 1.796	4, 1.782	4, 1.766	4, 1.745	4, 1.742
μ, mm^{-1}	3.409	3.361	3.335	3.306	3.266	3.261
F(000)	2796					
Crystal size, mm^3	0.3 x 0.25 x 0.25					
θ range, °	3.19 – 26.32	3.18 – 26.32	2.82 – 26.32	3.16 – 26.32	3.14 – 26.32	3.14 – 26.32

Reflections collected	11202	11041	11979	11639	12440	11855
Reflections unique [R_{int}]	5197 [0.0181]	5273 [0.0157]	5318 [0.0189]	5371 [0.0156]	5440 [0.0159]	5447 [0.0198]
Completeness to θ	0.998	0.998	0.998	0.998	0.998	0.998
Number of parameters	322					
Goodness-of-fit on F^2	1.027	1.013	1.028	1.003	1.041	1.046
Final R_1, wR_2 [I>2σ(I)]	0.0213, 0.0481	0.0228, 0.0513	0.0234, 0.0494	0.0250, 0.0551	0.0264, 0.0535	0.0287, 0.0538
R_1, wR_2 (all data)	0.0254, 0.0503	0.0292, 0.0548	0.0308, 0.0528	0.0344, 0.0602	0.0389, 0.0590	0.0433, 0.0632
$\Delta\rho_{max}$ / $\Delta\rho_{min}$, e·Å^{-3}	0.432 / -0.695	0.370 / -0.530	0.359 / -0.448	0.405 / -0.441	0.369 / -0.438	0.471 / -0.414
CCDC code	2182503	2182504	2182505	2182506	2182507	2182508

Table S2. Crystal data and structure refinement parameters for compound **2**.

Temp., K	100	240	300	353	403	423
Empirical formula	$[C_{22}H_{26}Br_2N_4O_2Mn]_2^{+}[ReCl_6]^{2-}$					
Molecular weight	1585.35					
Crystal system, space group	monoclinic, $C2/c$					
a, Å	18.1265(3)	18.2200(2)	18.2974(4)	18.3684(4)	18.4095(6)	18.4455(4)
b, Å	15.8310(3)	15.9405(2)	16.0015(3)	16.0336(5)	16.0709(6)	16.1065(4)
c, Å	18.1065(3)	18.1738(2)	18.2295(4)	18.2340(5)	18.2638(6)	18.2993(4)
β, °	90.133(2)	90.056(1)	90.054(2)	90.375(2)	90.520(3)	90.602(2)
Volume, Å^3	5195.8(2)	5278.3(1)	5337.4(2)	5370.0(2)	5403.3(3)	5436.3(2)
Z, $\rho_{calc.}$, g/cm^3	4, 2.027	4, 1.995	4, 1.973	4, 1.961	4, 1.949	4, 1.937
μ, mm^{-1}	6.238	6.141	6.073	6.036	5.999	5.962
F(000)	3084					
Crystal size, mm^3	0.3 x 0.25 x 0.2					
θ range, °	2.82 – 26.32	2.81 – 26.32	3.15 – 26.32	3.14 – 26.32	2.80 – 26.32	3.12 – 26.32

Reflections collected	10922	12092	11778	12234	12444	11622
Reflections unique [R_{int}]	5273 [0.0196]	5351 [0.0212]	5411 [0.0190]	5453 [0.0182]	5494 [0.0207]	5525 [0.0259]
Completeness to θ	0.998	0.997	0.998	0.998	0.998	0.998
Number of parameters				322		
Goodness-of-fit on F^2	1.035	1.071	1.034	1.021	1.022	1.011
Final R_1, wR_2 [$I>2\sigma(I)$]	0.0256, 0.0544	0.0251, 0.0560	0.0272, 0.0584	0.0290, 0.0613	0.0337, 0.0662	0.0382, 0.0771
R_1, wR_2 (all data)	0.0319, 0.0569	0.0332, 0.0595	0.0406, 0.0633	0.0450, 0.0679	0.0557, 0.0755	0.0703, 0.0877
$\Delta\rho_{max}$ / $\Delta\rho_{min}$, e·Å^{-3}	0.584 / -1.519	0.852 / -1.052	0.806 / -1.188	0.861 / -1.113	0.914 / -1.144	0.558 / -1.325
CCDC code	2182509	2182510	2182511	2182512	2182513	2182514

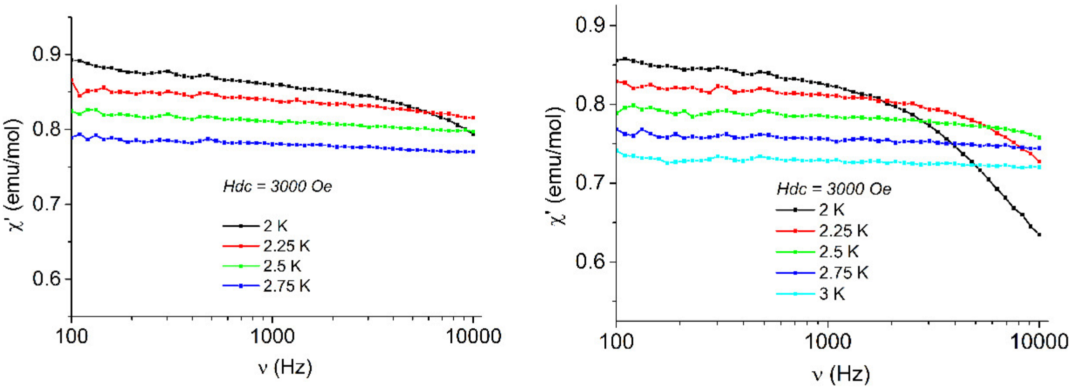

(**a**) (**b**)

Figure S7. The frequency dependency of the χ' for complexes **1** (a) and **2** (b) at temperatures of 2-3 K under the magnetic field of *Hdc* = 3000 Oe.
